# An Address Delivered to the Members of the Worcestershire Natural History Society, on the Opening of the Worcestershire Museum, September 15, 1836

**Published:** 1837-07

**Authors:** 


Art. VI.—*An Address delivered to the Members of the Worcestershire Natural History Society, on the Opening of the Worcestershire Museum, September* 15, 1836. By Charles Hastings, m.d. f.g.s. —*London*, 1837. 8vo. pp. 57.

Knowing how much medical science, strictly so called, is improved and adorned by the knowledge derived from the study of the collateral sciences, we wish to lose no opportunity of pointing out to our readers, (particularly our younger readers,) the great importance of attending to these in prosecuting their studies. Natural history, in particular, has especial claims to their notice, as every branch of it is calculated to supply, not merely amusement of the most interesting, varied, and exhaustless kind, but information of the greatest value in regard to the knowledge and practice of medicine. The present discourse, the production of a man to whom the medical profession in this country is under the greatest obligations, is well calculated to inspire a love for this delightful science: we, therefore, strongly recommend it to our readers. It contains an exposition of the happy results flowing from the establishment of institutions for the cultivation of natural history; remarks on the advantages and pleasures derived from the study of this science; and a succinct account of the progress of natural history from the earliest times. It is earnest and eloquent, and displays at once the good taste and good feeling of its excellent author.

